# Supplementary material for: Graphene-edge dielectrophoretic tweezers for trapping of biomolecules
Source: Nat Commun. 2017 Nov 30;8:1867. doi: 10.1038/s41467-017-01635-9 (PMC5709377; doi:10.1038/s41467-017-01635-9)
Supplement: Supplementary file 2 — Additional Supplementary Files [file 41467_2017_1635_MOESM2_ESM.pdf]

Description of Additional Supplementary Files

File Name: Supplementary Movie 1
Description: Graphene-edge dielectrophoretic tweezers for manipulation of polytryrene beads (190 nm diameter).

File Name: Supplementary Movie 2
Description: Frequency dependence of graphene-edge DNA localization using 10 kbp DNA at 10 pM concentration (4x real time).
